# Determination of the Membrane Transport Properties of Jurkat Cells with a Microfluidic Device

**DOI:** 10.3390/mi10120832

**Published:** 2019-11-29

**Authors:** Tianhang Yang, Ji Peng, Zhiquan Shu, Praveen K. Sekar, Songjing Li, Dayong Gao

**Affiliations:** 1 Department of Fluid Control and Automation, Harbin Institute of Technology, Harbin 150001, Heilongjiang, China; yangth@hit.edu.cn; 2 Mechanical Engineering, University of Washington, Seattle, WA 98195, USA; pjosh730@uw.edu (J.P.); zq.shu@wsu.edu (Z.S.); pksekar@uw.edu (P.K.S.); 3 School of Mechanical and Materials Engineering, Washington State University, Everett, WA 98201, USA

**Keywords:** microfluidics, Jurkat cell, cell membrane permeability, osmotic behavior, two-parameter transport formalism

## Abstract

The Jurkat cell is an immortalized line of human acute lymphocyte leukemia cells that is widely used in the study of adoptive cell therapy, a novel treatment of several advanced forms of cancer. The ability to transport water and solutes across the cell membrane under different temperatures is an important factor for deciding the specific protocol for cryopreservation of the Jurkat cell. In this study we propose a comprehensive process for determination of membrane transport properties of Jurkat cell. using a novel microfluidic controlled single cell-trapping system. The osmotic behavior of an individual Jurkat cell to water and dimethyl sulfoxide (DMSO), a commonly used cryoprotective agent (CPA), under constant temperature, was recorded under a microscope utilizing the modified microfluidic system. The images of the Jurkat cell under osmotic change were processed to obtain a relationship between cell volume change and time. The experimental results were fitted using a two-parameter transport numeric model to calculate the Jurkat cell membrane permeability to water and DMSO at room temperature (22 °C). This model and the calculated parameters can help scientists optimize the cryopreservation protocol for any cell type with optimal cryoprotective agents and cooling rate for future experiments.

## 1. Introduction

Freezing is lethal to most living systems, yet cells can endure long periods of storage at low temperatures such as −196 °C for centuries [1,2]. Addition of cryoprotective agents (CPAs) could greatly affect the osmotic behavior of cells and eliminate cryoinjuries during freezing. A standard cryopreservation protocol involves 7 main steps [3]: pre-processing, CPA addition, cooling to subzero temperatures, storage at low temperature, thawing, dilution and CPA removal, and post-processing. During the cooling, thawing, addition and removal of CPAs, the changes in the intracellular and intercellular conditions can be lethal to cells [4,5,6,7,8]. Based on Peter Mazur’s “Two-Factors” hypothesis, slow cooling rates will lead to severe cell dehydration as water leaves the cell, whereas fast cooling rates can also lead to fatal “ice injury” to the cells due to there being insufficient time for water transport across the membrane leading to intracellular ice formation (IIF) [9].

Osmotic injury and intracellular ice formation injury are both fatal to cells during cryopreservation and they are firmly related to the transmembrane water transportation [10]. The study of transmembrane mass transfer properties of cell membrane will help researchers to optimize cryopreservation protocols to achieve the best cell viability. The most important properties to be determined are cell membrane permeability coefficient to water (hydraulic conductivity, Lp) and cell membrane permeability coefficient to CPAs (Ps).

Researchers found evidence that mass transfer properties of a biological membrane could be influenced and/or regulated by many factors, such as: cell age [11], attractive optical potential [12], specificity of membrane proteins [13] and shape of aquaporins on the cell membrane [14]. Several devices have been developed to quantify Lp and Ps for different cells and were thoroughly reviewed by McGrath [15] and Zhao [16]. The most common technique is using a microfluidic channel to observe the single cell osmotic volume change under a microscope. Using data processing and a mathematical model for the transmembrane material transport, the related cell membrane properties can be obtained by parameter fitting.

In order to stably capture and observe one or a few cells at specific locations, several devices have been designed utilizing multiple strategies. These cell-trapping devices can generally be categorized into two types: contact and non-contact. Contact type includes methods using micropipette, perfusion chamber and holding block. Non-contact type includes methods utilizing acoustic tweezers, magnetic field, electric field and flow field [17]. Gao [18] developed a micro-perfusion system using a micropipette to hold a mouse oocyte by applying a small negative pressure on its zona pellucida. The mouse oocyte could be perfused directly into a solution of interest using another micropipette. This method could switch the extracellular solution immediately, but it could only be applied to limited type of cells. McGrath’s group [19] developed a micro-diffusion system that consists of a solution flow channel, a sample chamber and a layer of porous membrane between the channel and the chamber. Cells can be stabilized in the chamber under the hydraulic pressure difference across the membrane. However, the porous membrane can be damaged during fabrication of such a sandwich structure and the concentration of extracellular solution may not be changed instantaneously. Tseng [20] developed a device by bonding a layer of PDMS (polydimethylsiloxane) with microfluidic channel onto a glass slide. The upper surface of the glass slide is treated by poly-D-lysine hydrobromide so that cells could adhere on it and then be observed and recorded. The disadvantage of this method is the requirement of rapid flow rate.

Guo [21] developed a 3D acoustic tweezer using surface acoustic wave generators to generate pressure nodes surrounding the experimental area and trap a single cell in a certain 3D space. This system could manipulate cells without physical contact; however, introducing high frequency sound wave (13 MHz) itself and the heat that might be generated by it could influence the state of cells. Di Carlo [22] developed a high-throughput automated microfluidic technology capable of probing single-cell deformability using inertial focusing at an intersection area to stretch cells in an extensional flow. In that system, cells were carried and measured while being entirely surrounded by fluid. Later, Fang [23] developed a dynamic microfluidic control system using the stagnation point in a flow field to trap individual cells at a similar intersection area. Based on precise flow control, this system could avoid cell damage and could switch extracellular solutions instantly. Although this design could give highly accurate results, manufacturing the multilayer microfluidic chip and operating such complex control system requires considerable cost and labor work.

Chalut [24] developed a optofluidic assay to confine single cells. Cells were squeezed into channels smaller than the average cell size under pressure gradient and images were taken while cells were entering, passing through as well as exiting the channel for deformation analysis. Pagliara [25,26] developed a device to investigate the physiology of viable but non-culturable (VBSN) cells with multiple dead-end narrow lateral microchannels connected to a main microchamber. This device could perform drug treatment on the single cell that filled in the lateral channel, but the solution switching took a considerably long time. Yobas [27,28] and Di Carlo [29] reviewed and summarized methods of cell separation and sorting in microfluidic systems among which weir, pillar, crossflow and membrane are four designs that belong to the microfilter method. The weir filter involves an individual barrier obstructing the flow path to isolate white blood cells from red blood cells in a given whole blood. Red blood cells can pass only through a narrow slit located on top of the barrier.

Chen [30], Di Carlo [31] and Heo [32] also developed several microfluidic devices separately using a PDMS microfluidic chip with a block inside the channel to trap cells. With a slow and steady flow inside the microfluidic channel, cells can be held at the block. Taking advantage of laminar flow at the micro scale, the trapped cells could be perfused into CPA solutions immediately and completely by changing solutions from the inlet of the channel. This type of design is easy to fabricate, operate and could be applied to cells of different sizes by modifying the size of the block and channel.

Jurkat cells are an immortalized line of human T lymphocyte cells of highly spherical shape with the diameter ranging from 10 to 16 μm, first derived from the peripheral blood of a 14-year-old boy suffering from T cell leukemia [33]. Jurkat cells can be transfected and can produce interleukin-2 (IL-2) [34,35]. Therefore, they can be used as the host to study T-cell receptor (TCR) signaling which are greatly related to adoptive cell therapy—a novel treatment of several advanced forms of cancer and many other researches on cancer treatments [36]. Jurkat cells are also useful for research of blood proto-oncogenes expression, apoptosis and cell survival (e.g., HIV) [37,38,39]. They can also be used for studies of differentiation [37,38,39,40]. Cryopreservation of Jurkat cells is very useful as researchers could rewarm and study the same batch of cells without mutation after a long storage period. Cryopreservation has a significant importance on saving the cost and maintaining the consistency of cell properties for researchers.

DMSO (dimethyl sulfoxide) is one of the mostly used cryoprotective agents nowadays. However, DMSO exposure can directly impact cellular function and growth to a level depending on type of cell, the stage of cell development and differentiation, the specific DMSO concentration, temperature and the duration of exposure as we discussed in our review paper [41]. In order to find out whether DMSO is the best choice for Jurkat cell cryopreservation and provide better protocols for Jurkat cell cryopreservation in the future, the Jurkat cell membrane permeability coefficients to water and DMSO were studied.

The advent of microfluidic techniques provide an ideal platform for microscale cell manipulation and allow further studies on cell-specific cell osmotic behaviors [16]. Utilizing a microfluidic controlled single cell-trapping device, experiments can be performed to record cell volume change versus time. A mass transfer model with constant temperature was built to obtain Lp and Ps utilizing the relationship between time and osmotic cell volume change. In our work, we optimized Chen’s design to observe cell volume change from an isotonic environment (297 mmol) to a commonly used CPA (10% DMSO, v/v) in 1× PBS (phosphate-buffered saline)) solution to determine Lp and Ps at room temperature. The whole system includes a microscope with a high-speed camera, a precisely controlled syringe pump and a microfluidic chip that is connected with the pump through a tubing. The microfluidic chip is made by bonding a polydimethylsiloxane, PDMS (Sylgard 184, Dow Corning Corporate, Auburn, MI, USA) layer onto a glass slide. The PDMS layer is a replica of a reusable master with desired micro structurers fabricated by soft lithography. The entire chip is transparent so that the camera under the chip could record the images of Jurkat cells inside the channel for later analysis. The volume of cells could be obtained by image and data processing using MATLAB (MathWorks, Natick, MA, USA) [42,43,44]. By parameter fitting, the relationship between cell volume change and time into related numerical models, Lp and Ps of Jurkat cell membrane could be quantified.

## 2. Materials and Methods

### 2.1. Source of Cells

Human acute lymphoblastic leukemia cells (Jurkat cells, Clone E6-1) used in the experiments were purchased from ATCC (American Type Culture Collection, Manassas, VA, USA). Cells were cultured in T25 flasks inside a 37 °C incubator with 5% carbon dioxide and proper humidity. A unit of the culture medium contains 450mL RPMI (the base medium, salts, buffer, sugars, etc., RPMI Medium 1640(1×), Life Technologies, Thermo Fisher Scientific Inc., Waltham, MA, USA), 50mL fetal bovine serum (FBS), 5mL Penicillin-streptomycin and 5mL L-glutamine.

### 2.2. Preparation of Cells 

On the day of the experiment, 2 mL cell suspension was collected from the flask. After counting with a cell counter (Countess II FL, Thermo Fisher Scientific Inc., Waltham, MA, USA), cell suspension was centrifuged at 500 *g* for 5 min and then resuspended with culture medium to make a density of 1 × 10^6^/mL. The experiments were finished within 3 hours to ensure the activity and viability of Jurkat cells. The size, morphology and viability of cells was also be evaluated by the cell counter.

### 2.3. Design and Fabrication of the Microfluidic Chip

Our design uses a block on the top of the microfluidic channel to stop the cells and keep the fluid flowing underneath the block. This block lowered the height of the microchannel at the trapping area. The manufacturing of a PDMS microfluidic chip with a block structure in the microchannel requires a mold with microstructure of different heights that was fabricated on a silicon wafer using multilevel soft lithography. The height of both the block and the channel can be modified according to the cell size of interest. In our study, since the isosmotic diameter of Jurkat cell usually ranges from 13–20 μm, we chose 5 μm as the height of the microchannel under the trapping block and 20 μm as height at other parts of the microchannel so that only one Jurkat cell would be trapped at the block vertically. Detailed fabrication steps of the mold and PDMS chip can be found in our previous work [30].

**Figure 1:**
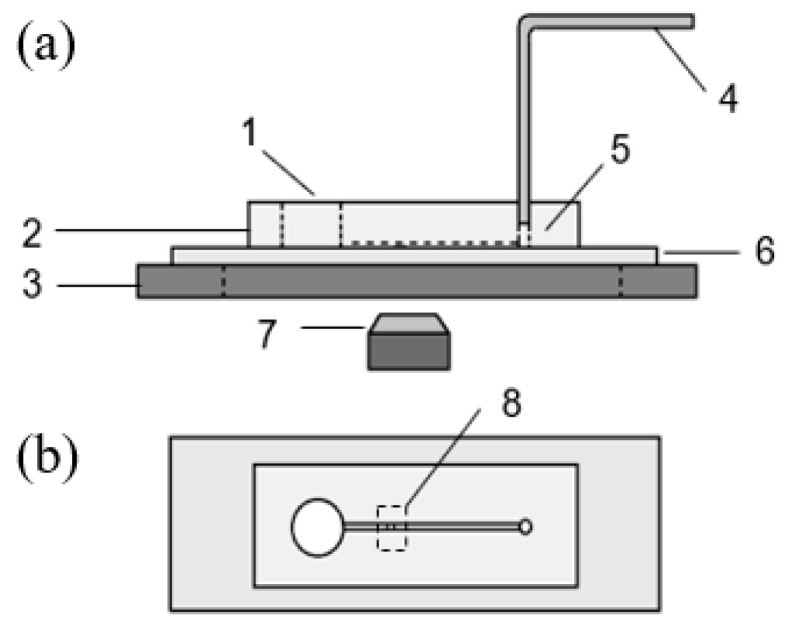
Sideview (**a**) and Top view (**b**) of the cell trapping system with a block structure: **1** medium solution reservoir(inlet); **2** polydimethylsiloxane (PDMS) microfluidic chip; **3** microscope holding stage; **4** tubing connected to syringe pump; **5** outlet; **6** substrate glass slide; **7** microscope lens and camera and **8** trapping area.

After peeling off the PDMS chip from the mold, a 1 mm hole was punched at one end of the channel to serve as the outlet and a 5 mm hole was punched at the other end near the block to serve as the inlet for the fluid flow, see Figure 1. A shorter distance between the inlet, blocking area and outlet was chosen to reduce the flow resistance to trap the cells more efficiently. A larger inlet reduces the influence of residual fluids while switching solutions and also reduces the pressure disturbance due to different liquid level. The PDMS chip was irreversibly bonded to a glass slide after oxygen plasma treatment using a plasma cleaner (PDC-32G, Harrick Plasma, Ithaca, NY, USA) under 18 W and 60–90 Pa for 60 s. The bonding performance of the chip could be enhanced by placing the bonded chip on a hot plate at 60 °C for 2 min; 1× PBS solution was added into the channel immediately after the surface treatment since it modifies the surface from hydrophilic to hydrophobic in a short time, and it can be much more difficult to fill the microchannel with liquid after that.

### 2.4. Setup of the Device and Operation Procedure

The whole device consists of an inverted microscope ((Nikon, Eclipse Te2000-s, Chiyoda, Japan), a camera (Phantom V310, Vision research, Wayne, NJ) with the resolution of 600 by 600 pixels, a PDMS-glass microfluidic cell trapping chip, a precisely controlled micro syringe pump (Cetoni GmbH, neMESYS, Korbussen, Germany) and its control system as shown in Figure 1.

The fluid, containing the prepared cells, was perfused into the inlet reservoir gently using a pipette and then sucked into the microchannel under negative pressure provided by a syringe pump at the outlet. One end of a tubing was connected to the matching connector of the precisely controlled micro syringe pump and the other end was pushed into the outlet hole of the microfluidic chip. The microscope was placed under the trapping area to record the cell volume change. 

**Figure 2:**
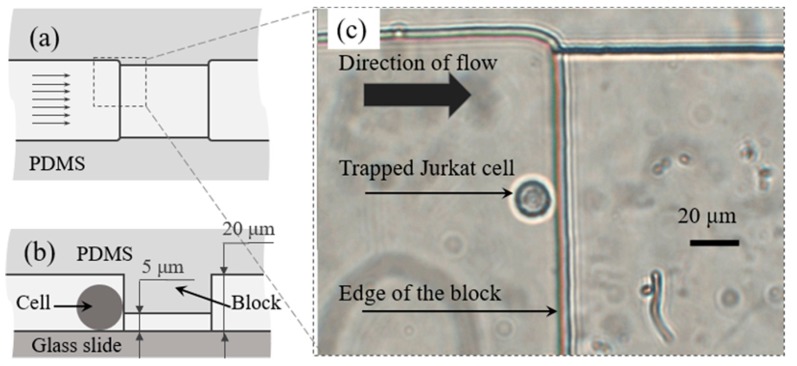
Schematic diagram showing top view (**a**) and side view (**b**) of trapping area in microfluidic channel, parallel arrows indicate the direction of the flow. A Jurkat cell was trapped by the trapping block and recorded by microscope (**c**).

Since PDMS is an elastic material, the connection between the chip and tubing was well sealed; 20–30 µL of cell suspension (cells in 1× PBS) was put into the inlet reservoir with a pipette and the syringe pump was set to provide a flow rate of 10–20 µL/h. The flow rate was then adjusted to 5–10 µL/h after several cells were stopped and trapped at the blocking area. As shown in Figure 2, one Jurkat cell in the flow coming from the left side was stopped by the block in the microchannel while the fluid went through under the block. A small low flow rate was used to avoid deformation of cells during the experiments, as cells can be deformed and pushed under the block. To switch the solution, the fluid residue was removed from the inlet reservoir gently and 20 µL 10% DMSO (v/v) in 1× PBS solution or other anisotonic medium was added into it using a pipette.

The camera was started to record the images of trapped cells right before the addition of CPA solution. The entire osmotic volume changing process under this circumstance usually happens within few minutes. A frame rate of 5 images per second was found to be optimal for our later parameter fitting work. 

### 2.5. Image Analysis and Data Processing 

**Figure 3:**
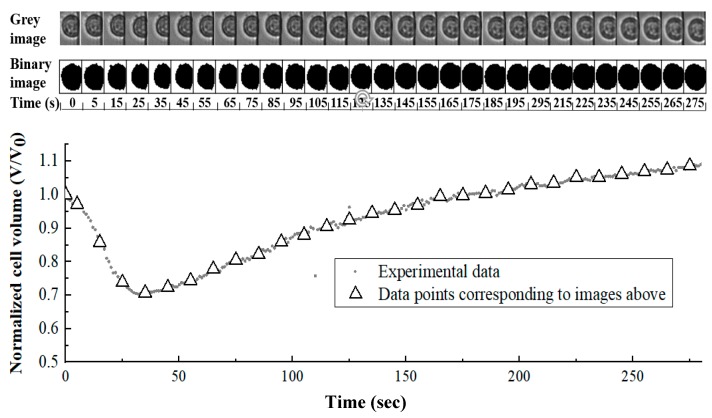
Grayscale images, binary images and quantified cell volume values of single Jurkat cell volume change after environment changed from isotonic solution to 10% (v/v) dimethyl sulfoxide (DMSO) in 1× phosphate-buffered saline (PBS).

In the circumstance of penetrating CPA addition (e.g., DMSO), the water loss from the cytoplasm is usually faster than CPA penetration into cells under chemical potential gradients, which depends on cell membrane property as well. As a result, the cell would appear to be shrinking at first and then swelling as the intercellular environment reached the equilibrium with the extracellular environment. In our study, this process was recorded by camera as shown in Figure 3.

Isolated cells in a better spherical shape on the raw images were cropped out in order to isolate single cell out of background interference. The cropped images were then converted from color to 8-bit gray scale images. Using MATLAB, a gray intensity threshold was chosen where the images were divided at the clear edge of cells based on gray intensity of pixels. The software could automatically divide and transform the images into small black and white square pixels referring the threshold so that the image of the cell would be distinguished from background. The number of pixels that belong to the cell part on the processed image was counted and then a mathematical relationship was built to transfer that number of pixels into actual cell volume (V) as well as cell surface area (A). Utilizing the same relationship, cell volume change while extracellular environment switching from isotonic solution into 10% (v/v) DMSO in 1× PBS solution under room temperature could be obtained.

### 2.6. Validation of Replacement Time between Different Media

Although the instantaneous step change of extracellular concentration has been claimed/assumed [15,18,45], the solution switch still takes some time. A coupled multi-physical model was built using COMSOL Multiphysics (COMSOL Inc., Palo Alto, CA, USA) to study the process of solution switch in the microchannel. Time-dependent osmolarity distributions in the microchannel were simulated and visualized [46].

Peclet number (*Pe*) was also used to interpret the phenomenon during the solution switch. It is defined as the ratio of the rate of advection of a physical quantity by the flow to the rate of diffusion of the same quantity driven by an appropriate gradient. The Peclet number in our platform is calculated as:

(1) $$Pe=\frac{UL}{D},$$

where *U* represents local linear flow velocity, *L* is the characteristic length, and *D* is the mass diffusion coefficient. The Pe during the experiments was around 20 which meant the diffusion at the boundary between two consecutive solutions is negligible. In the other word, we can assume that the cell was immediately switched from isotonic solution to hypertonic solution (10% DMSO in our study).

### 2.7. Mathematical Model of Cell Volume Change to Cryoprotective Agent (CPA) Addition

The cell volume change can be expressed by the mass flux across cell membrane. For the circumstances with only non-permeable solutes being added into the solution, the water flux across the cell membrane that is related to chemical potential gradient can be expressed as [1]:

(2) $$\frac{dV_{c}\left(t\right)}{dt}=L_{p}\cdot A\cdot \left(C_{n}^{i}-C_{n}^{e}\right)\cdot R\cdot T,$$

where $V_{c}$(*t*) is cell volume (um^3^) at time t (min), $L_{p}$ is the cell membrane permeability to water (μm·min^−1^·atm^−1^), *A* is the average surface area of a single cell (um^2^), $C_{n}^{i}$ is the intracellular osmolality (Osm/kg), $C_{n}^{e}$ is the extracellular osmolality (Osm/kg), R is universal gas constant (0.08207 atm·L/(mol·K)), *T* is absolute temperature (K, in our study *T*, equals to 22 °C, the room temperature). While the extracellular solution is switched from isotonic to hypertonic, loss of cytoplasmatic water will lead to cell shrinkage until intracellular osmolality reaches an equilibrium with the extracellular osmolality. Since the only reason of cell volume change is the loss of water, $C_{n}^{i}$ can be expressed as:

(3) $$C_{n}^{i}=C_{0}\cdot \left(\frac{V_{0}-V_{b}}{V-V_{b}}\right),$$

where $C_{0}$ is isotonic osmolality (Osm/kg, for blood and body fluid is approximately 0.3 Osm/kg, which is also our initial intracellular osmolality), $V_{0}$ is the isotonic cell volume (μm^3^), $V_{b}$ is the osmotically inactive cell volume (μm^3^) that could be determined by the Boyle van’t Hoff plot.

Although a few researchers previously considered the interaction between solvent and solute into the numerical model of transmembrane mass transfer while they both transport through a common channel specifically [28,47], discoveries in molecular biology indicate that, in natural biological membranes, co-transport of water and penetrating solute is often unlikely or negligible [26]. As a result, for the numerical model of CPA addition with both penetrating and non-penetrating solutes, cell volume change can be expressed as:

(4) $$\frac{dV_{c}\left(t\right)}{dt}=\frac{dV_{s}\left(t\right)}{dt}+L_{p}\cdot A\cdot \left(C^{i}-C^{e}\right)\cdot R\cdot T.$$

The penetrating CPA flux across cell membrane can be expressed as:

(5) $$\frac{dN_{s}\left(t\right)}{dt}=P_{s}\cdot A\cdot \left(C_{s}^{e}-C_{s}^{i}\right),$$

where $V_{s}\left(t\right)$ is the intracellular CPA volume (um^3^) at time t, $C^{i}$ and $C^{e}$ are intra- and extracellular osmolality including both penetrating and non-penetrating solutes (Osm/kg), $N_{s}\left(t\right)$ is the mole number of intracellular CPA at time t, $P_{s}$ is the cell membrane permeability (cm/min) to penetrating CPA solute, $C_{s}^{e}\;\mathrm{and}\;C_{s}^{i}$ are extra- and intracellular osmolality of penetrating CPA solutes (Osm/kg). 

$L_{p}$ and $P_{s}$ can be determined by parameter fitting the differential Equations (4), (5) with data from former experiments using software Mathematica (Wolfram Research, Champaign, IL, USA).

## 3. Results

### 3.1. Obtaining Osmotically Inactive Cell Volume (V_b_) of Jurkat Cell

Osmotic inactive cell volume is one of the most important parameters in the mathematical model as shown in Equation (3). It indicates the cell volume when the osmolality of extracellular solution approaches infinity. The osmotically inactive cell volume (*V*_b_) of a Jurkat cell can be determined by measuring the average Jurkat cell volume in several saline solutions with different osmolality and then doing the Boyle van’t Hoff plot with the cell volume and the reciprocal of the osmolality of the solution.

In order to obtain the cell volume, cell morphology in several hypotonic and hypertonic PBS solutions were recorded using a microscope. Each image taken has 5–9 cells on it. The raw images were processed and the average cross-section area of cells was given utilizing Image J (National Institute of Health, Bethesda, MA, USA). Average cell diameters could then be obtained with a simple calculation. PBS solutions in different osmolality could be obtained by mixing high concentration PBS solution with different amounts of de-ionized (DI) water. Cell suspension were added in a series of PBS solutions with different osmolality PBS solutions and the mixture was hold for 5 min before measuring in order to reach osmotic equilibrium. The osmolality of the mixture was tested using a vapor pressure osmometer (MODEL 5520, WESCOR Vapro, Logan, UT, USA).

**Figure 4:**
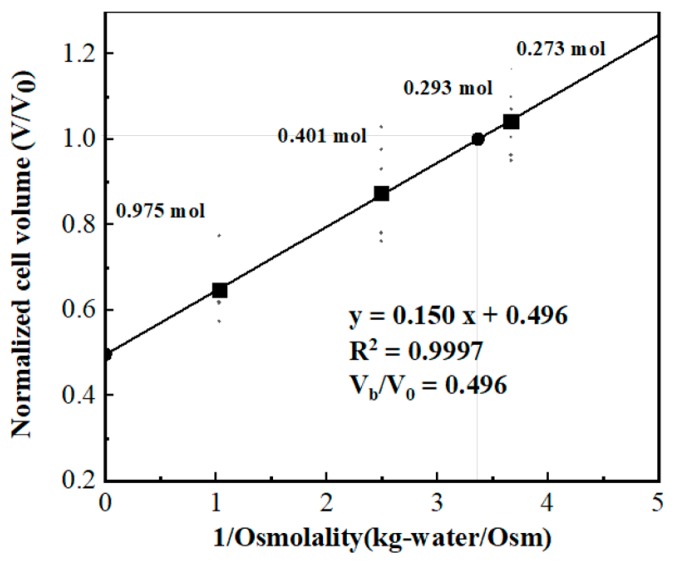
Measurement of osmotic inactive cell volume (*V*_b_).

In this study, we measured the cell equilibrium volume under 173, 401 and 975 mmol/kg environments, with data collected from at least 6 sets. According to the Boyle van’t Hoff equation, a linear line can be obtained by linearly fitting cell volume that normalized to isotonic cell volume to the reciprocal of corresponding osmolality, and the intercept of that fitting line at the y axis equals the ratio of the osmotically inactive volume (*V*_b_) to isotonic cell volume (*V*_0_). As the Boyle van’t Hoff plot shown in Figure 4, for Jurkat cell, *V*_b_/*V*_0_ equals 0.496 in our study.

### 3.2. Solution Switching in the Microchannel

**Figure 5:**
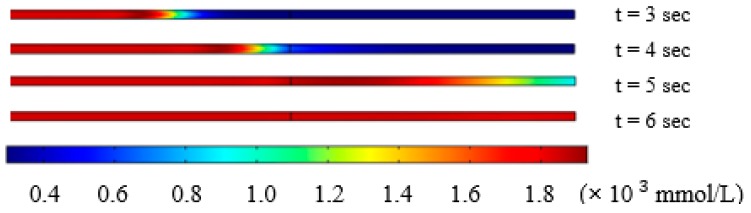
Osmolarity change inside the microchannel.

In our study, the solution in the microchannel was switched from cell suspension (297 mmol/L) to 10% DMSO (v/v) in 1× PBS solution (1823 mmol/L). As indicated in the methods section, the osmolarity distribution inside the microchannel at 3, 4, 5 and 6 s after changing solution at the inlet of the device at left side was simulated using COMSOL, as shown in Figure 5. The trapping block is located at the middle line of the microchannel in Figure 4.

**Figure 6:**
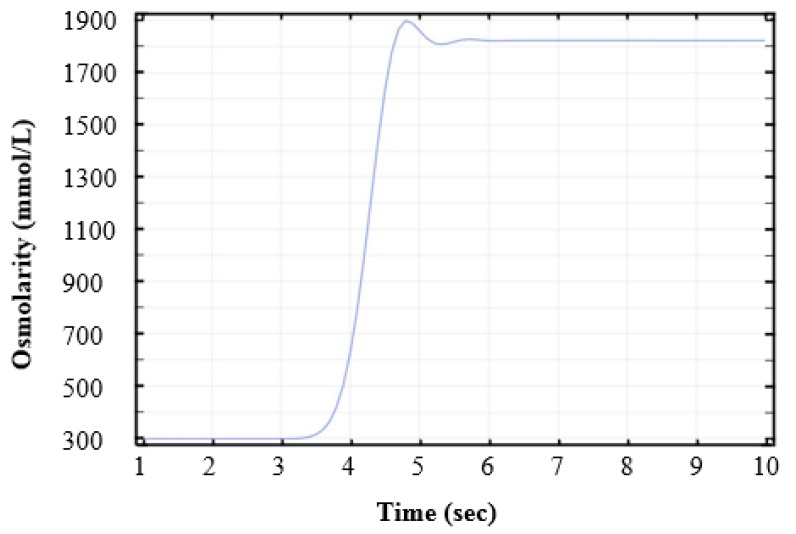
Time-dependent osmolarity change at the block.

As a result of physical phenomena that happened at the micro scale, CPA diffusion developed rather slowly during the switching process. The simulation result of osmolarity change to time at the block was recorded in Figure 6. Solution switching at the trapping block from isotonic to hypertonic environment was completed within 1.5 s, as shown in Figure 6. The results indicate that extracellular environment changed in a very short time and we could assume that solution switching completed instantly.

### 3.3. Cell Membrane Permeability Coefficient to Water (Lp) and CPAs (Ps) by Curve-Fitting

**Figure 7:**
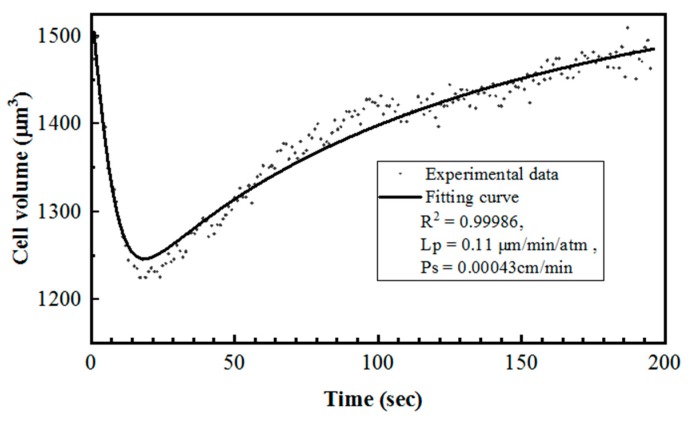
Parameter fitting of Jurkat cell volume change during switch from 1× PBS to 10% DMSO (v/v) in 1× PBS at room temperature.

In order to find the cell membrane properties Lp and Ps, the data sets of cell volume change to time were used as V_c_ and t, respectively, to undertake parameter fitting in coupled differential Equations (4) and (5). As shown in Figure 7, both water loss and CPA transport happened at a rather fast speed during the initial part of the change. The Jurkat cell membrane permeability coefficient to water and to DMSO in 22 °C were found by our method to be 0.148 ± 0.051 μm·min^−1^·atm^−1^ and 0.00034 ± 0.00014 cm/min (n = 8).

## 4. Discussion

The Jurkat cell is an immortalized line of human acute lymphocyte leukemia cells that has been used in many studies such as T-cell activation and T-cell receptor signaling. Those studies have great importance to virus and cancer treatment. Several Jurkat cell lines are available from commercial companies nowadays. However, in order to keep the consistency of cells for research purposes and save the cost of materials, the cryopreservation of Jurkat cells is still significant. During the optimization of the cryopreservation protocols, including the choice of CPA solution and optimal cooling rate, the measurement of its cell membrane permeability to water and CPA such as DMSO is one of the most important procedures. With the help of those parameters, researchers can find out how the cell volume and intracellular osmolality would change during different situations using numerical simulation. A proper choice of both CPA and cryopreservation protocol would protect cells from osmotic injury during CPA addition/removal and leave minimum yet necessary water inside the cell to avoid lethal intracellular ice crystallization. Therefore, in order to measure the Lp and Ps of Jurkat cells, a simple, reliable microfluidic platform was developed by our group. With our device, a suspended Jurkat cell would flow into the microchannel under negative pressure, be stopped by a trapping block in the channel, perfused by the CPA solution and undergo volume change that would be recorded by a camera.

In our design, the instantaneity of the solution switch at the cell-trapping stage is extremely important for the following data analysis and the accuracy of calculated cell membrane properties. To verify this assumption, the transport process of CPA solution in the microfluidic channel was simulated with COMSOL. Based on the experimental settings, the diffusion of CPA was relatively small compared with the convection of the fluid flow. The unwanted osmolarity gradient of CPA at the cell-trapping block would only exist for a short time. Based on the simulation results, the time was around 1.5 s or less for the replacement of isotonic solution by the target hypertonic solution, which is comparable to the previous study [30]. The result was also qualitatively proved by the relatively high value of Pe number for the flow in the channel.

The parameter fitting result of Lp in our study was 0.14 μm·min^−1^·atm^−1^ and Ps (to DMSO) was 0.00034 cm/min in 22 °C. For reference, former results have shown Lp to be 0.38 μm·min^−1^·atm^−1^ and Ps as 0.00049 cm/min for rat basophilic leukemia cell [30], Lp as 0.18 μm·min^−1^·atm^−1^ and Ps as 0.00046 cm/min for human granulocyte [48], and Lp as 0.295 μm·min^−1^·atm^−1^ and Ps as 0.00098 cm/min for human vaginal mucosal macrophage [49]. A considerably convincing result was determined by the parameter-fitting process in this study compared with other results.

The method combining the experimental setup and two-parameter transport model could be used as a standard investigating approach for other cell types. Using the microfluidic device, the cell membrane permeability to water and CPA were determined which will assist cell cryopreservation, including the selection of optimal CPA, the optimization of CPA addition and removal, and the prediction of the optimal cooling rate for the cells. For example, the current work shows that the measured Ps value to DMSO for Jurkat cells was relatively low compared to that for many other cells presented in previous papers. From this study DMSO, a widely used CPA for cryopreservation, may not be the optimal choice of CPA for Jurkat cells. Further research to investigate other CPAs for Jurkat cells will be conducted.

## 5. Conclusions

Through simulation and image-processing analysis, the cell membrane permeability of Jurkat cells to water and CPA (DMSO) was determined with our microfluidic device. The measured properties could be used to generate optimal cryopreservation protocols for Jurkat cells. Potential research can also be inspired by the results, such as exploring optimal CPAs (high permeability and low toxicity) and optimizing the cooling process for Jurkat cells.
